# Epigenetics Regulates Antitumor Immunity in Melanoma

**DOI:** 10.3389/fimmu.2022.868786

**Published:** 2022-05-25

**Authors:** Yuhan Chen, Xiuli Yi, Ningyue Sun, Weinan Guo, Chunying Li

**Affiliations:** ^1^ Department of Dermatology, Xijing Hospital, Fourth Military Medical University, Xi’an, China; ^2^ School of Basic Medical Sciences, Fourth Military Medical University, Xi’an, China

**Keywords:** epigenetics, antitumor immunity, melanoma, immunotherapy, DNA methylation

## Abstract

Melanoma is the most malignant skin cancer, which originates from epidermal melanocytes, with increasing worldwide incidence. The escape of immune surveillance is a hallmark of the tumor, which is manifested by the imbalance between the enhanced immune evasion of tumor cells and the impaired antitumor capacity of infiltrating immune cells. According to this notion, the invigoration of the exhausted immune cells by immune checkpoint blockades has gained encouraging outcomes in eliminating tumor cells and significantly prolonged the survival of patients, particularly in melanoma. Epigenetics is a pivotal non-genomic modulatory paradigm referring to heritable changes in gene expression without altering genome sequence, including DNA methylation, histone modification, non-coding RNAs, and m^6^A RNA methylation. Accumulating evidence has demonstrated how the dysregulation of epigenetics regulates multiple biological behaviors of tumor cells and contributes to carcinogenesis and tumor progression in melanoma. Nevertheless, the linkage between epigenetics and antitumor immunity, as well as its implication in melanoma immunotherapy, remains elusive. In this review, we first introduce the epidemiology, clinical characteristics, and therapeutic innovations of melanoma. Then, the tumor microenvironment and the functions of different types of infiltrating immune cells are discussed, with an emphasis on their involvement in antitumor immunity in melanoma. Subsequently, we systemically summarize the linkage between epigenetics and antitumor immunity in melanoma, from the perspective of distinct paradigms of epigenetics. Ultimately, the progression of the clinical trials regarding epigenetics-based melanoma immunotherapy is introduced.

## Background

Melanoma is among the most aggressive forms of skin cancer, the worldwide incidence of which has been continuously increasing. The malignant transformation of epidermal melanocytes contributes to the occurrence of melanoma, which proliferates rapidly and can easily metastasize throughout the body *via* lymphatic and blood vessels (1, 2). While melanoma at an early stage can be cured by surgical resection, metastatic melanoma spreading to distant spots, in particular to pivotal organs like the lung, liver, and brain, would be difficult to treat and greatly threatens the lifespan of patients (3). In 2021, there are approximately 106,110 new cases of cutaneous melanoma and 7,180 cases of mortality arising from this disease in the United States, accounting for approximately 5.6% of all new cancer cases emerging and 1.2% of all cancer-related deaths, respectively (4).

Previous accumulating evidence has revealed that the primary risk factors of melanoma mainly include family history, multiple moles, fair skin, AND UV radiation, and genetic alteration is considered to be related to all these risk factors (5). To be specific, the risk loci in *CDKN2A* are identified in about 40% of familial melanomas (6). In addition, individuals carrying *MC1R* variants, especially those with red hair color, fair skin, and poor tanning ability, might have a higher risk of melanoma (7). Of note, melanoma possesses the highest mutational burden among all types of cancers, which is largely attributed to UV radiation. UVB might cause a C-to-T transition in the 3′ base of the pyrimidine. Another classic UV characteristic mutation is the G-to-T transformation caused by UVA-related oxidative damage (1, 2, 8). Advances in the understanding of the genetic mutations operating in melanoma pathogenesis have led to revolutionary progress in targeted therapies that interfere with mutation drivers like *BRAF* (9). Apart from targeted therapy, the invigoration of antitumor immunity *via* the blockade of immune checkpoints like PD-1 or PD-L1 has also been ubiquitously employed in melanoma treatment since 10 years ago, which has unprecedentedly optimized tumor control. Although the treatment like the combination of BRAF inhibitor and MEK inhibitor or monoclonal anti-PD-1 antibody has increased the 5-year survival from less than 5% to approximately 30% (4), the prognosis of patients with advanced melanomas remains unoptimistic. Therefore, a more comprehensive understanding of the molecular mechanisms underlying melanoma pathogenesis is necessary for the generation of more innovative and effective therapies and more clinical options for the treatment of melanoma.

Recently, epigenetic modification, referring to heritable changes in gene expression without an alteration in the genome sequence, has attracted more attention in clarifying the pathogenesis of melanoma (10). The core regulatory mechanism is the covalent modifications of either histone tails or nucleosome complexes that modulate chromatin structure and gene expression (11). During the whole process of melanoma development, epigenetic profiles of tumor cells are dynamically altered, especially concerning DNA methylation and histone acetylation (12, 13). Moreover, the dysregulation of epigenetics might prominently affect various biological characteristics of tumor cells, including cell proliferation, cell plasticity and stemness, cell invasion and migration, cell metabolism, and tumor immunology (14–18). Therefore, the mechanistic understanding of these processes would help to extensively unveil melanoma pathogenesis and provide more accessible and druggable choices for developing alternative therapeutic approaches. As epigenetics in tumor immunology is greatly implicated in not only the regulation of tumor cell immunogenicity but also the antitumor capacity of immune cells (19), targeting epigenetics is sure to be a potential strategy to reinforce the efficacy of immunotherapy. For example, the usage of pan-HDAC inhibitor leads to the upregulated expression of PD-L1 and PD-L2 in melanoma, which can help to increase the treatment response to anti-PD-1 antibody and prolong the survival of preclinical mice (20). Moreover, in a preclinical mouse model of melanoma, low-dose 5-aza combined with an anti-CTLA-4 antibody has been proved to be more effective in controlling tumor growth than monotherapy (21). Therefore, efforts have been increasingly paid to clarifying the linkage between epigenetics and antitumor immunity in melanoma, which might bring about innovative therapeutic approaches for melanoma treatment.

## Tumor Microenvironment and Antitumor Immunity

Melanoma is the most lethal skin cancer with relatively high proliferative capacity and aggressiveness, which largely results from various immunosuppressive mechanisms that often work in concert to help tumor cells evade innate and adaptive immune detection and destruction (22–25). The characteristic of immune evasion depends on the interaction between tumor cells and the surrounding tumor microenvironment (TME). Aside from large amounts of tumor cells, the TME is a multicomponent and complex network consisting of cancer-associated fibroblasts (CAFs), endothelium, keratinocytes, adipocytes, and various types of immune cells (26, 27). While previous studies emphasized investigating the intrinsic characteristics of tumor cells, the complicated and decisive interplay between tumor cells and immune cells has gradually attracted more attention. Of note, the capacity of tumor-infiltrating immune cells to eliminate tumor cells, termed antitumor immunity, can be prominently reprogrammed to contribute to the progression of melanoma (28–30).

From a Darwinian perspective, evading immune-mediated destruction is a critical step of tumor evolution, and the immune system is one of the strongest selective pressures during tumorigenesis. In the early stage, immune cells play their due functions by inducing the apoptosis of transformed cells, producing antitumor cytokines, or inducing cytotoxic reactions according to their intrinsic characteristics. Later, tumor cells that are insensitive to immune cell-mediated elimination and of high proliferative capacity would be selected to forwardly facilitate tumor progression (22, 31). The effective antitumor immunity during the early phase of tumor development could be generally divided into adaptive immunity and innate immunity. For adaptive immunity, upon the recognition of tumor cell antigen, surrounding dendritic cells (DCs) would migrate to lymph nodes and present antigens to immature T cells to contribute to their subsequent full activation. CCR7 receptor expressed by DCs is essential for this antigen-presentation process in melanoma (32, 33). There are three main types of T lymphocytes, namely, effector cells (or cytotoxic cells), helper cells, and regulatory cells (34). To be specific, CD8^+^ T effector cells are responsible for recognizing antigens proposed by antigen-presenting cells through MHC class I molecules and play a cytotoxic role by directly inducing the apoptosis of melanoma cells *via* releasing perforin (32, 35). In addition, CD4^+^ T helper cells (Th) usually bind to antigen-presenting cells (APCs) through MHC class II protein complexes and can differentiate into several types of immune cells with different capacities (36). For example, Th1 cells are capable of producing IFN-γ to obtain the inhibitory effect on tumor cells (37). There are also large amounts of tumor-associated macrophages (TAMs) existing in or around tumors, which are recruited by chemokine CCL2 released by either cancer cells or stromal cells, to participate in extracellular matrix degradation, tumor cell migration, and angiogenesis (38). In addition, it is found that the number of neutrophils gradually increases during tumor progression and can be polarized into type N1 with antitumor activity or type N2 with an immunosuppressive effect. During the early stage of tumor progression, type N1 neutrophils dominate in TME to mediate the killing of melanoma cells, whereas type N2 neutrophils dominate in the late stage to support tumor extravasation (38, 39). In some circumstances, when a subset of tumor cells have the capacity to resist the killing effect of T cells, NK cells would work properly to play a compensatory role in recognizing and eliminating these tumor cells. Nevertheless, melanoma cells can also escape NK cell-mediated lysis by suppressing the expression of major NK receptors that are associated with immune function, including NKp30, NKp44, and NKG2D (40, 41). Previous investigations have paid much attention to therapeutic approaches aiming to activate the cytotoxic function of CD8^+^ T cells. Excitingly, several recent therapeutic strategies for inhibiting melanoma growth have focused specifically on activating the antitumor activity of naive or undifferentiated inherent immune cells within TME, including macrophages, NK cells, and DCs, all of which are primary cells to produce non-specific cytotoxicity to target melanoma cells (41). Through the activation of NK receptors (NKG2D, NKp30, NKp46, and DNAM-1) on the surface of melanoma cells, NK cells can be independently activated to eliminate melanoma cells (41, 42). In addition, macrophages can also participate in adaptive immunity by fulfilling the activation of CD8^+^ effector T cells and mediating the generation of memory immune populations involved in long-term remission.

Although melanoma is one of the most immunogenic types of tumors that are putatively easier to be recognized and eliminated by the immune system, the plasticity of melanoma cells allows them to adapt to the cytotoxic TME. Tumor cells can acquire different immunogenic characteristics as well as the ability to produce immunomodulatory molecules, which in turn influences the activation of immune cells or the composition of immune infiltration within the tumor (43, 44). For example, apart from PD-L1, melanoma cells can express a relatively high level of IDO-1 that exerts an inhibitory effect on the cytotoxicity of NK cells (45, 46). In addition, melanoma cells can secrete chemokines to recruit immunosuppressive regulatory T cells (Tregs), which can effectively block the cytotoxicity of effector T cells by releasing inhibitory cytokines (47–49). Moreover, melanoma cells also secrete granulocyte-macrophage colony-stimulating factor (GM-CSF) or IL-6 and transport exosomes loaded with microRNAs to recruit and transform bone marrow myeloid-derived suppressor cells (MDSCs) that inhibit the activity of effector T cells through various mechanisms (50). In addition, tumor-derived secretory molecules like VEGF-A contribute to the downregulation of endothelial cell adhesion molecules such as ICAM-1, thereby inhibiting T-cell adhesion to endothelial cells and subsequent infiltration in the tumor. Aside from this, tumor-secreted VEGF-A, prostaglandin E2, and IL-10 can induce FasL expression in endothelial cells, directly leading to CD8^+^ T-cell death (51). More importantly, tumor cells suppress the expression of chemokines, such as CXCL9 and CXCL10, by increasing promoter methylation, thereby limiting the necessary directional cues for T-cell infiltration (52). It has also been proposed that tumor cells secrete Galectin-3 to sequestrate IFN-γ in the extracellular matrix and limit IFN-γ-mediated chemokine production and immune cell scheduling. Hence, melanoma cells take advantage of their high immunogenicity and a variety of epigenetic signaling pathways to achieve immune escape, which greatly challenges melanoma treatment and medication (53). In summary, the immune system plays a key role as the first-line defense against melanoma, and the understanding of the interplay between tumor cells and the immune system provides cellular and molecular bases of immunotherapy to generate effective antitumor immunity.

## Epigenetics in Melanoma Antitumor Immunity

Different from genetic mutations and variations, epigenetics refers to heritable changes in gene expression without the alteration of the genome sequence. DNA methylation, histone modification, non-coding RNA regulation, and m^6^A mRNA methylation are four main paradigms of epigenetic modifications that can re-shape chromatin structure and regulate gene expression mainly *via* covalent modification of histone tail or nucleosome complex (54, 55). Previous studies have emphasized the role of dysregulated epigenetics in the intrinsic characteristics of melanoma cells, revealing the close linkage between epigenetics and melanoma cell biology (56, 57). However, more and more reports are now extending the network of epigenetics-driven tumor immunology in melanoma.

### The Crosstalk Between DNA Methylation and Melanoma Immunology

#### Brief Introduction of DNA Methylation and Its Role in Melanoma Cell Biology

As the most well-studied epigenetic modification in cancer, DNA methylation is the process of 5-methylcytosine (5-mC) formation, which is dynamically regulated by DNA methyltransferases (DNMTs) and ten-eleven translocation (TET) family members that are responsible for the transfer of methyl groups to cytosine residues and DNA demethylation *via* the oxidative catalysis of 5-mC to form 5-hmC (58). The downregulation of 5-hmC induced by the reduction of isocitrate dehydrogenase 2 (IDH2) and TET family members is a valuable epigenetic marker with relatively high diagnostic and prognostic significance in melanoma (59). Quite a few genes implicated in cell differentiation, epithelial–mesenchymal transformation, PI3K/mTOR signaling, and cell metabolism are differentially methylated in the promoter regions between melanomas and benign nevus (60, 61). Genome-wide DNA methylation analysis also unveils the epigenetic heterogeneity of melanoma metastases in specific organs (62). Moreover, several studies have demonstrated the engagement of DNA methylation in the regulation of specific oncogene or tumor suppressors in melanoma. For example, Field et al. showed that canonical tumor suppressor *BAP1* is hypermethylated in uveal melanoma. In addition, integrative analysis reveals that SOX9 is methylated and lowly expressed in the highly proliferative group, and SOX9 overexpression results in decreased proliferation but increased invasion of melanoma cells (15). Therefore, DNA methylation plays a versatile role in mediating various aspects of melanoma cell biology.

#### DNA Methylation in Immunologic Characteristics of Tumor Cells

In addition to tumor cell biology, the dysregulation of DNA methylation plays a critical role in regulating the immunologic characteristic of melanoma cells from various dimensions, including antigen presentation, inflammatory response pathways, and immune checkpoint molecules.

The effective recognition and targeting by tumor-infiltrating lymphocytes highly rely on the presence and levels of HLA class I antigen expression on the surface of tumor cells. The loss of the HLA class I antigen could help tumor cells to escape this killing effect, which to some extent results from the hypermethylation of the related genes (63, 64). This regulatory relationship is also supported by the finding that the treatment with DNA methylation inhibitor 5′-aza-2′-deoxycytidine (DAC) greatly contributes to the restoration of cell surface expression of HLA class I antigens and potentiates tumor cell recognition by MAGE-specific cytotoxic T lymphocytes (CTLs) (65). It is tempting to speculate that the hypermethylation-induced lack of HLA class I expression is the cause of the impaired response to vaccination, which provides a new route of escape from immune recognition. Similarly, Radosevich et al. also provided evidence that class II MHC molecules driven by the gene encoding the class II transactivator (CIITA) could also be significantly restored after the treatment with DAC in uveal melanoma cells, especially when stimulated by IFN-γ (66). Therefore, DNA methylation is a critical paradigm for the regulation of HLA antigens in melanoma cells.

The disordered DNA methylation in melanoma cells can also regulate inflammatory response pathways to modulate the immunologic characteristics. Chiappinelli et al. unveiled that the application of DNMT inhibitor prominently induces the expression of endogenous retrovirus (ERV) genes and the cytosolic sensing of double-stranded RNA (dsRNA) that cause a type I interferon response. Bioinformatics analysis displays that high viral defense signature expression in tumors is significantly associated with the durable clinical response of immunotherapy. Notably, DNMT inhibitor treatment sensitizes tumors to anti-CTLA-4 therapy in a preclinical melanoma model, which is attributed to the re-activation of the interferon response pathway (67, 68). Additionally, a recent study carried out by Falahat et al. with the employment of genome-wide DNA methylation profiling has noted that the promoter hypermethylation of cGAS and STING genes leads to their coordinated transcriptional silencing and contributes to the widespread impairment of the STING signaling function in clinically relevant human melanoma. Pharmacologic inhibition of DNA methylation could reinstate functional STING signaling, which is effective to restore STING signaling to improve the antigenicity of tumor cells through the upregulation of MHC class I molecules, facilitating the recognition and killing of melanoma cells by cytotoxic T cells within TME (18). Thus, epigenetic reprogramming of tumor cell-intrinsic STING function by modulating DNA methylation might be a promising approach to increase the efficacy of T cell-based immunotherapy. Moreover, it has been revealed that the deletion of TET2 in murine melanoma significantly reduces chemokine expression and TILs, thus enabling tumors to evade antitumor immunity and resist anti-PD-L1 immunotherapy. Conversely, to potentiate TET activity by systematic injection of its co-factor ascorbate or vitamin C could increase chemokine and TILs, leading to enhanced antitumor immunity and anti-PD-L1 efficacy and extending the lifespan of tumor-bearing mice. This investigation also suggests TET activity as a biomarker for predicting the efficacy and patient response to anti-PD-1/PD-L1 therapy (69).

Beyond the effect on tumor intrinsic pro-inflammatory pathways, a series of recent investigations demonstrate the involvement of DNA methylation in regulating the expressions of multiple immune checkpoint molecules like PD-L1, PD-L2, CTLA-4, LAG3, TIM-3, and Galectin-9. For example, the integrated genomic analysis identified that global DNA methylation regulates PD-L1 expression in melanoma. The inhibition of global methylation by decitabine increases PD-L1 expression in melanoma cells (70). Furthermore, PD-L2 expression is also under the control of DNA methylation. The methylation status of *PD-L2* gene can be used to predict the progression-free survival of melanoma patients who have received anti-PD-1 antibody treatment (71, 72). Similar to PD-L2, the methylation status of *CTLA-4* is also inversely correlated with its mRNA level in melanoma tissue. The evaluation of CTLA-4 methylation provides paramount information for selecting patients more likely to respond to immunotherapy, especially to anti-CLTA-4 antibody treatment (73, 74). Moreover, several studies also illustrate the effect of DNA methylation on the mRNA level of LAG3, TIM-3, and Galectin-9 (75, 76).

In aggregate, the immunophenotypes of melanoma cells are largely influenced by DNA methylation, which is thus involved in regulating antitumor immunity and the response to immunotherapy (**Table 1**).

**Table 1:** The crosstalk between DNA methylation/histone modification and melanoma immunology.

Class of epigenetic alteration	Aspect of melanoma immunology	Detailed underlying mechanism	References
**DNA methylation**	**Tumor cell immunologic characteristics**	Promoter hypermethylation of the HLA class I and II molecules leads to downregulation of HLA antigens	(63–66)
		Promoter hypermethylation suppresses endogenous retrovirus (ERV) genes, the cytosolic sensing of double-stranded RNA (dsRNA), and the downstream type I interferon response	(67)
		Promoter hypermethylation of cGAS-STING genes induces widespread impairment of the STING signaling	(18)
		TET2 deficiency significantly reduces chemokine expression and TILs, enabling tumors to evade antitumor immunity and to resist anti-PD-L1 immunotherapy	(69)
		Promoter hypermethylation of PD-L1, PD-L2, CTLA-4, LAG3, TIM-3, and Galectin-9 induces their downregulation	(70–76)
	**Antitumor capacity of immune cells**	Methylation pattern resembles stromal and leukocyte cells, and an overexpressed immune signature	(77)
		Gp96 engages conventional and plasmacytoid dendritic cells (pDCs) by altering global methylation *via* CD91	(78)
		DNA hypomethylating agent promotes CD8^+^ T-cell infiltration and cytotoxic function	(79)
**Histone modification**	**Tumor cell immunologic characteristics**	Histone deacetylase inhibitors promote the expressions TAP1, TAP2, LMP2, LMP7, and Tapasin and facilitate antigen processing and presentation.	(80, 81)
		HDAC inhibitor induces prominent upregulation of PD-L1, increasing the efficacy of anti-PD-1 antibody	(20)
		LSD1 ablation increases tumor immunogenicity by upregulating ERV transcripts and suppressing RNA-induced silencing complex	(82)
		Histone methylase EZH2 induces the suppression of IFN-γ signature	(83)
	**Antitumor capacity of immune cells**	Histone deacetylase SIRT1 impedes the differentiation of Th17 cells *via* the reduction of STAT3 acetylation	(84)
		HDAC6 inhibition results in downregulation of Th2 transcription factor GATA3, upregulation of the Th1 transcription factor T-Bet, accumulation of central memory phenotype T cells, reduced exhaustion-associated phenotypes, and enhanced killing in mixed lymphocyte reactions	(85)
		SIRT2 overexpression suppresses the infiltration and function of natural killer cells in tumor microenvironment	(86)
		Targeting LSD1 phosphorylation effectively induces IFN-γ/TNF-α-expressing CD8^+^ T-cell infiltration into the tumors	(87)
		EZH2-mediated histone H3 methylation of HIF1α in macrophages reprograms the immune suppressive microenvironment to the facilitative one	(88)

#### DNA Methylation in Antitumor Capacity of Immune Cells

The alteration of DNA methylation also displays a prominent effect on the features and functions of immune cells within TME. DNA methylation profile analysis of melanoma tissues has identified a subgroup of patients with methylation patterns resembling stromal cells and leukocytes, an overexpressed immune signature, and improved survival rates (77). Furthermore, the global methylation profile is regarded as an independent indicator of tumor cell immune evasion and response to immune checkpoint inhibition in melanoma (89, 90). The analysis of methylation profile then more specifically focuses on tumor-infiltrating immune cells with the method to extract methylation of immune cell type-specific genes from genome-wide methylation arrays. Intriguingly, multiple immune methylation clusters are related to patients’ survival. Moreover, low-dimensional projection based on immune cell type-specific methylation reveals the grouping of the solid tumors in line with melanoma immune methylation clusters rather than tumor types (91). These results highlight the close linkage between immune methylation signature and immune cell function.

In terms of the mechanistic study, heat shock protein gp96 engages conventional and plasmacytoid DCs (pDCs) through CD91, with the involvement of global DNMT-dependent epigenetic modifications, to change the protein expression within these antigen-presenting cells (78). As a result, pDCs upregulate neuropilin-1 to enable the long-term interactions of pDCs with Treg cells, thereby enhancing the suppression of Th1 antitumor immunity. Moreover, a recent report pointed out that pharmacological agent leading to DNA hypomethylation in CD8^+^ T cells vastly regulates activation-related transcriptional networks, which helps to potentiate effector cytokine production and antitumor activity. Therefore, *in vivo* employment of DNA hypomethylating agent promotes the infiltration of CD8^+^ T cells and suppresses tumor growth *via* CD8^+^ T cell-dependent cytotoxicity in melanoma (79). Taken together, the antitumor capacity of immune cells within TME is tightly regulated by DNA methylation in melanoma, which underpins developing therapeutic strategy of combining DNA methylation-regulatory agent and immune checkpoint blockades (**Table 1**).

### Histone Modification and Antitumor Immunity in Melanoma

#### An Overview of Histone Modification and Its Role in Melanoma Cell Biology

Chromatin flexibly switches from a dense heterochromatin state with poor transcriptional activity to a relaxed euchromatin state with high transcriptional activity. Histone modification usually occurs at the N-terminal “tail” of histones, displaying prominent effects on shaping chromatin structure and regulating gene expression (92). Typical histone modification paradigms include acetylation, methylation, phosphorylation, and ubiquitination. Specifically, histone acetylation refers to the addition of acetyl-to-lysine residues, which is reversely regulated by histone acetyl transferases (HATs) and histone deacetylation enzymes (HDACs) (93). Furthermore, histone methylation occurs at lysine or arginine residues at specific sites of histone H3 and H4, dynamically modulated by lysine-specific demethylase (LSD) and a variety of methyl-transferases (94). A recent systematic epigenomic profiling analysis found frequent loss of histone acetylations and H3K4me2/3 on regulatory regions proximal to cancer-regulatory genes implicated in melanoma-driving pathways, suggesting the extensive participation of histone acetylation and methylation in melanoma (12, 13). The expression levels of histone acetylation-modulatory enzymes are frequently dysregulated in melanoma (95–97). Our group has previously reported that histone deacetylase SIRT6 plays a bimodal role in melanoma *via* regulating IGF1R-Akt axis and the related autophagy (98). In addition, histone acetyltransferase P300 directly binds to the promoter region of melanocytic-lineage factor MITF to enhance mitochondrial oxidative phosphorylation and thereby melanoma growth (99). Additionally, the role of histone methylation in melanoma biology has also been well illustrated. H3K9 demethylases LSD1 and JMJD2C jointly contribute to melanoma progression by facilitating the transcription and expression of E2F target genes (100). Intriguingly, H3K9 methylation regulated by SETDB1, LSD1, and JMJD2C appears to play quite opposite roles in the occurrence and development of melanoma (12, 13). While SETDB1-induced increase of H3K9 methylation exerts a prominent carcinogenic effect in established tumors (101, 102), the restoration of H3K9 methylation by targeting LSD1 and JMJD2C abolishes the malignant transformation from melanocytes to melanoma driven by oncogenic mutations (103). Therefore, histone methylation plays a bimodal role in melanoma carcinogenesis and development, and targeting histone methylation should conform to the stage status in melanoma therapy.

#### Histone Modification in Tumor Cell Immunogenicity

Apart from tumor cell biology, the dysregulation of histone modification also exerts prominent influences on the immunologic characteristic of melanoma cells, including antigen presentation, immune checkpoint molecules, and inflammatory pathways.

The initial investigation on the role of histone modification in tumor cell immunogenicity was centered on the expression of genes related to MHC class I or class II antigen presentation. Khan et al. firstly reported that histone deacetylase inhibitors could induce the expression of molecules like TAP1, TAP2, LMP2, LMP7, and Tapasin, which are involved in antigen processing and presentation *via* the MHC class I pathway in melanoma cells (80). The facilitative role of HDAC inhibitor trichostatin A on the expression of antigen processing machinery is forwardly confirmed by another study (81). Of note, the *in vivo* antitumor effect of TSA is found to be entirely dependent on the alteration of immunogenicity of tumor cells, as the tumor-suppressive effect does not occur in immune-deficient mice (81). However, the pharmacological toxicity experiment reveals that the IC_50_ of pan-HDAC inhibitor panobinostat is much lower in peripheral blood mononuclear cells (PBMCs) than that in melanoma cells, indicating that HDAC inhibitor is cytotoxic in PBMCs at concentrations much lower than those required for melanoma antitumor activity (104). Therefore, a more detailed analysis of HDAC family molecules in melanoma tumor immunology was further carried out. Woan et al. reported that HDAC6 mediates the expression of tumor-associated antigens and MHC class I expression. Targeting HDAC6 helps to achieve a pronounced delay of *in vivo* melanoma tumor growth that partly depends on an intact immunity (105).

In addition to the effect on antigen presentation, HDAC inhibitor also prominently regulates immune checkpoint expression (e.g., PD-L1) in the tumor. Mechanistically, HDAC inhibitor induces rapid upregulation of histone acetylation of the *PD-L1* gene to elicit enhanced and durable gene expression, which thereby helps to increase the efficacy of anti-PD-1 antibody (20). Intriguingly, histone methylation is another important paradigm implicated in tumor cell immunologic characteristics. The ablation of LSD1 increases tumor immunogenicity by upregulating ERV transcripts and suppressing the RNA-induced silencing complex, which is responsible for the diminished resistance to anti-PD-1 immunotherapy in melanoma (82). According to this notion, targeting LSD1 is of great translational potential in optimizing the efficacy of immunotherapy. Moreover, the expression of histone methylase EZH2 is highly associated with suppressed IFN-γ signature in the tumor. EZH2 inhibitors may be most effectively targeted to immunologically cold melanoma to achieve both the direct cytotoxicity and increased immune responses in the context of checkpoint inhibitor immunotherapy (83).

Taken together, dysregulated histone acetylation and methylation in melanoma regulate tumor cell immunogenicity through manipulating various approaches including antigen presentation, immune checkpoint molecules, and inflammatory pathways.

#### Histone Modification in Immune Cell Function Within Tumor Microenvironment

Accumulating evidence has revealed that histone modification is also central to regulating the function of different immune cells in TME, including T cells, NK cells, and macrophages.

Histone acetylation and methylation were previously proved to impact the antitumor activity of different subtypes of T cells. Specifically, the activation of histone deacetylase SIRT1 might impede the differentiation of Th17 cells *via* reducing STAT3 acetylation and relevant secretion of pro-angiogenic factors, thus restraining melanoma growth (84). Furthermore, while pan-HDAC inhibitor displays toxicity on immune cells, specific inhibition of HDAC6 by ACY-1215 results in the downregulation of the Th2 transcription factor GATA3, upregulation of the Th1 transcription factor T-Bet, accumulation of central memory phenotype T cells, reduced exhaustion-associated phenotypes, and enhanced killing in mixed lymphocyte reactions. Therefore, the observation that HDAC6-selective inhibitor augments T-cell immune properties in melanoma patients provides a rationale for translational investigation of their potential clinical efficacy (85). Additionally, Knox et al. proved that HDAC6 inhibitor facilitates the transition of the TME from “cold” to “hot” and profoundly synergizes with immune checkpoint blockade therapies in controlling tumor growth. Of note, the combined effect is associated with enhanced infiltration of immune cells, increased central and effector T-cell memory, and significantly reduced pro-tumorigenic M2 macrophages within TME (106). Similarly, histone methylation also regulates the function of infiltrating T cells. It is reported that nuclear LSD1 phosphorylated at serine 111 (nLSD1p) is enriched in PD-1^+^CD8^+^ T cells from resistant melanoma patients. Targeting the LSD1p nuclear axis effectively confers the infiltration of IFN-γ/TNF-α-expressing CD8^+^ T cells into the tumor, which is further augmented by combined immunotherapy. In addition, nLSD1p is regulated by the key T-cell exhaustion transcription factor EOMES in dysfunctional CD8^+^ T cells, and nLSD1p regulates EOMES nuclear dynamics *via* demethylation/acetylation switching of critical EOMES residues. Accordingly, there is a positive feedback loop consisting of LSD1 and EOMES in impairing the antitumor capacity of CD8^+^ T cells (87).

The dysregulation of histone modification also affects the function of alternative types of immune cells in melanoma. Systemic overexpression of SIRT2 promotes melanoma progression in immune-competent mice by suppressing the infiltration and function of NK cells in TME. Pharmacological inhibition of SIRT2 potentiates NK cell-dependent antitumor immunity and the growth of allograft melanoma (86), which might be of translational implication in increasing the efficacy of immunotherapy. Moreover, EZH2-mediated histone H3 methylation of HIF1α in macrophages leads to the silence of HIF1α expression and reprograms the immune suppressive microenvironment to the facilitative one, which alleviates the tumor burden and prolongs the overall survival of mice implanted with tumor (88).

Taken together, histone modification simultaneously affects tumor cell immunogenicity and the function of immune cells within TME, so as to mediate tumor immunology mainly through these two approaches (**Table 1**).

### NcRNA and m^6^A RNA Methylation in Melanoma Immunology

#### An Overview of NcRNA and m^6^A RNA Methylation in Melanoma Cell Biology

In addition to DNA methylation and histone modification, non-coding RNA and m^6^A RNA methylation are also critical types of epigenetic modification. The expression profiles of microRNAs, lncRNAs, and circRNAs in melanoma have been extensively studied and are found to be associated with a variety of tumor hallmarks (107–110). Studies of miRNAs in melanoma initially focused on their roles as potential biomarkers (111). For example, the level of either serum or tissue miR-16 is highly associated with the American Joint Committee on Cancer (AJCC) stage and the prognosis of melanoma patients (112). Thereafter, the function of miRNAs in angiogenesis, metastasis niche formation, and T-cell dysfunction within TME has also received extensive attention recently. For example, Pencheva et al. uncovered a convergent and cooperative miRNA network consisting of miR-1908, miR-199a-5p, and miR-199a-3p, which drive the metastasis and angiogenesis in melanoma (113). In addition, miR-30b/30d promotes the metastatic behavior of melanoma cells by directly targeting the GalNAc transferase GALNT7, leading to reduced immune cell activation and recruitment (114). Of note, the biological functions of some specific non-coding RNAs are highly correlated with lineage-specific factors in melanoma. In particular, the sequestration of miR-16 by the mRNA of pigmentation-associated enzyme TYRP1 promotes tumor growth (115, 116). Moreover, a recent annotated lncRNA SAMMSON is regulated by melanocytic-specific transcriptional factor SOX10, and the deficiency of SAMMSON is capable of restraining tumor progression and increasing the efficacy of MAPK-inhibition targeted therapy (109, 117, 118).

In addition to non-coding RNAs, m^6^A RNA methylation is another critical chemical modification found in mRNA and non-coding RNAs in eukaryotic cells (119). Jia et al. discovered that the downregulation of the m^6^A level is a characteristic of ocular melanoma and indicates a poor prognosis (120). However, the increased m^6^A RNA methylation of BACE2 induces its upregulation, which accelerates the tumorigenesis of ocular melanoma (121). These two reports indicate the paradoxical role of m^6^A methylation in ocular melanoma. In uveal melanoma, the expression of the main m^6^A regulatory enzyme METTL3 is found to be significantly increased. The knockdown of METTL3 prominently suppresses cell proliferation, colony formation, migration, and invasion *via* the downregulation of c-Met (122), indicating that METTL3 is a promising target for the treatment of uveal melanoma.

#### The Crosstalk Between NcRNA/m^6^A RNA Methylation and Melanoma Immunology

The roles of ncRNAs in melanoma biology have been well elucidated. Recently, some investigations have focused on clarifying the role of ncRNAs in melanoma immunology, as well as the underlying mechanism. Through the transcriptional analysis of the process responsible for the conversion of monocytes into MDSCs mediated by melanoma extracellular vesicles (EVs), a set of microRNAs (miR-146a, miR-155, miR-125b, miR-100, let-7e, miR-125a, miR-146b, and miR-99b) is found to be markedly associated with the function of MDSCs and the resistance to treatment with immune checkpoint inhibitors in melanoma patients (123). Forwardly, Mastroianni et al. reported that miR-146a controls the immune response in melanoma TME. MiR-146a level is significantly increased in melanoma tissue. Furthermore, T cells isolated from miR-146a-knockout mice exhibit higher expression of miR-146a-targeted gene STAT1 and STAT1-regulated cytokine IFN-γ. Interestingly, the combined inhibition of PD-1 and miR-146a is proved as a promising therapeutic approach to enhance the antitumor immune response elicited by checkpoint therapy (124). In terms of CD8^+^ T cell-dependent tumor immunology, the senescence and exhaustion of T cells are the major barriers toward successful cancer immunotherapy. MiR-155 is identified to enhance the antitumor function of CD8^+^ T cells by restraining T cell senescence and functional exhaustion through epigenetic silencing of drivers of terminal differentiation. Mechanistically, miR-155 enhances polycomb repressor complex 2 (PRC2) activity indirectly by promoting the expression of the PRC2-associated factor Phf19 through the downregulation of the Akt inhibitor, Ship1 (125). Martinez-Usatorre et al. then elucidated the mechanism responsible for the increased expression of miR-155 in tumor-infiltrating CD8^+^ T cells and demonstrated that high miR-155 expression in tumor-infiltrating CD8^+^ T cells may be a surrogate marker of the relative potency of *in situ* antigen-specific CD8^+^ T-cell response (126). Apart from the role of intrinsic miRNAs within CD8^+^ T cells, CD8^+^ T cells can also internalize exosomes from melanoma cells in TME, which induces the downregulation of T-cell response through decreased T-cell receptor (TCR) signaling and diminished cytokine and granzyme B secretion. Therefore, miRNAs in melanoma-derived exosomes also contribute to tumor immune evasion and could be a valuable therapeutic target (127).

**Figure 1:**
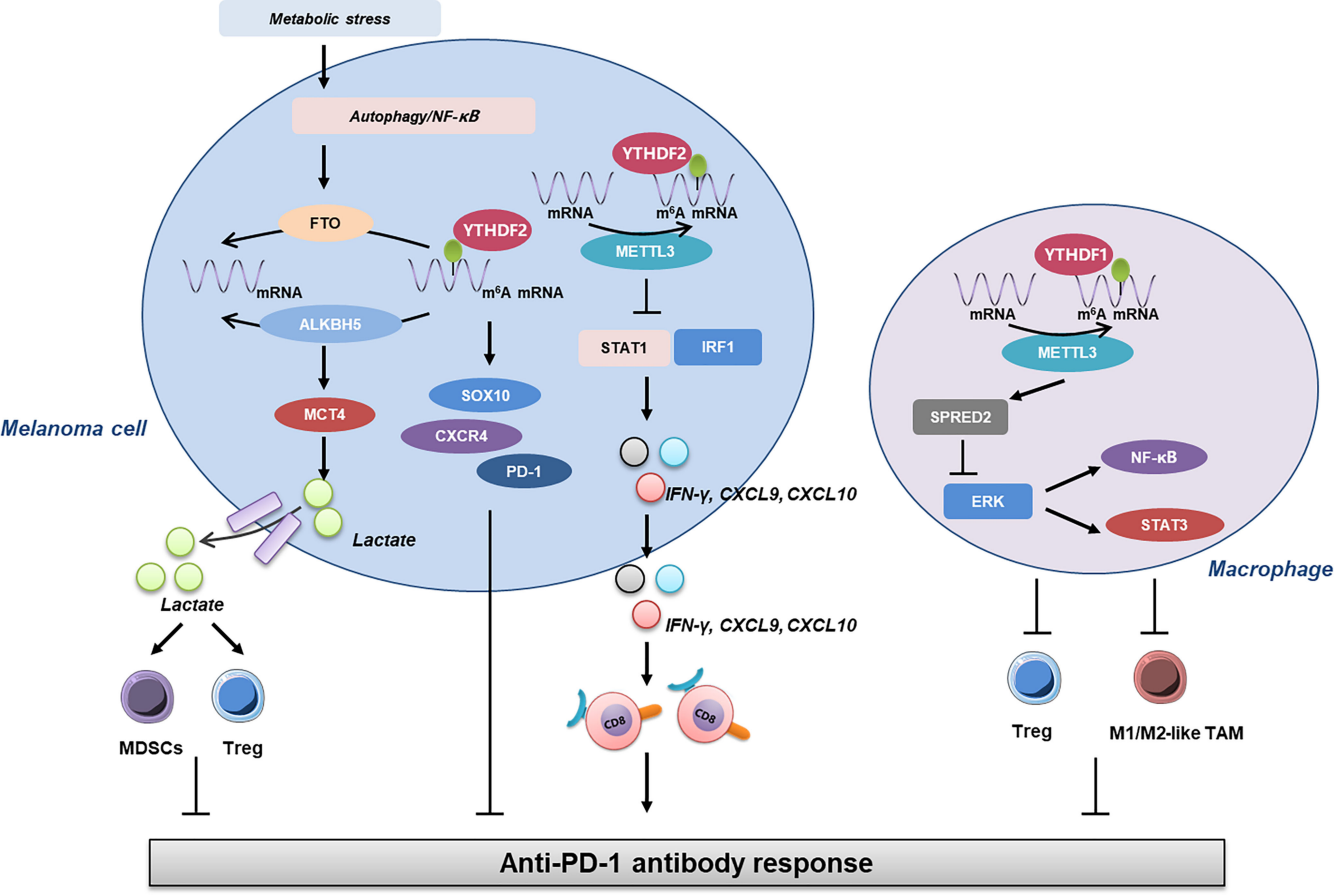
The linkage between m^6^A RNA methylation and antitumor immunity in melanoma. Under metabolic stress, the expression of FTO is induced under the control of autophagy and NF-κB, which contributes to the increase of m^6^A RNA methylation and subsequent upregulation of SOX10, CXCR4, and PD-1, leading to impaired response to anti-PD-1 antibody. Furthermore, ALKBH5 modulates MCT4 expression and lactate content in TME, so the composition of tumor-infiltrating Tregs and MDSCs is altered to affect the outcome of anti-PD-1 immunotherapy. In addition to this, METTL3 exerts its role in tumor immunology in either melanoma cells or macrophages. The deficiency of METTL3 in melanoma cells promotes the expressions of STAT1 and IRF1, resulting in the increase of IFN-γ, CXCL9, and CXCL10, which are critical for the infiltration and activation of CD8^+^ T cells. On the other hand, METTL3 in macrophages can impact the activation of ERK and downstream NF-κB and STAT3 *via* SPRED2, which then regulates the infiltration of Tregs and M1/2-like TAM and ultimately the response to anti-PD-1 antibody. FTO, fat mass and obesity-associated protein; TME, tumor microenvironment; Tregs, regulatory T cells; MDSCs, myeloid-derived suppressor cells.

Additionally, several recent reports have revealed the close linkage between m^6^A RNA methylation and antitumor immunity in melanoma (**Figure 1**). Yang et al. reported that under metabolic starvation stress, fat mass and obesity-associated protein (FTO) is prominently induced under the control of autophagy and the NF-κB pathway in melanoma. The genetic knockdown of FTO induces the increase of m^6^A methylation in the critical pro-tumorigenic melanoma cell-intrinsic genes including PD-1, CXCR4, and SOX10, which results in an increased RNA decay through the m^6^A reader YTHDF2. Therefore, the deficiency of FTO can help to sensitize melanoma cells to IFN-γ stimulation and increase the efficacy of anti-PD-1 immunotherapy in mice bearing melanoma xenograft tumor, which is associated with the activation of adaptive immunity (128). This study takes the lead in unveiling the crosstalk between m^6^A RNA methylation and tumor cell immunogenicity in melanoma. Then, the role of another m^6^A demethylase ALKBH5 is elucidated in melanoma immunology. In particular, ALKBH5 modulates Mct4/Slc16a3 expression and lactate content in TME, so that the composition of tumor-infiltrating Tregs and MDSCs is altered. Of note, the *ALKBH5* gene mutation and expression status of ALKBH5 in melanoma tissues are highly correlated with the response to immunotherapy in patients, indicating that ALKBH5 is not only a therapeutic target but also a reliable biomarker in predicting the efficacy of immunotherapy (129). Moreover, the depletion of methyltransferases METTL3 and METTL14 in tumor cells is found to prominently induce the activation of the IFN-γ-STAT1-IRF1 signaling axis through the stabilization of the STAT1 and IRF1 mRNA *via* YTHDF2. This regulatory effect leads to the elevated secretion of IFN-γ, CXCL9, and CXCL10, as well as the enhanced infiltration of cytotoxic CD8^+^ T cells within TME, ultimately resulting in potentiated response to anti-PD-1 antibody treatment. Notably, there is a negative correlation between METTL3/METTL14 and STAT1 in tumor tissues of 59 patients. In this case, METTL3 and METTL14 are also potential therapeutic targets in anticancer immunotherapy (130). Furthermore, the role of METTL3 in melanoma immunology is also attributed to its effect on tumor-infiltrating macrophages. METTL3-depleted macrophages were observed to reshape the TME *via* the enhancement of M1- and M2-like TAM and Treg infiltration in a transgenic mouse model with genetic ablation of *METTL3* in myeloid cells. This effect is caused by the impairment of YTHDF1-mediated translation of SPRED2, which induces subsequent activation of NF-κB and STAT3 through the ERK pathway, thus leading to precipitated tumor growth and metastasis. Intriguingly, the therapeutic efficacy of PD-1 checkpoint blockade is robustly attenuated in *METTL3*-deficient mice, indicating METTL3 as a potential therapeutic target for tumor immunotherapy, including in melanoma (131). Combined, these reports highlight that m^6^A RNA methylation is an important paradigm of epigenetic modification involved in the regulation of tumor immunology in melanoma, from the perspective of either tumor cell immunogenicity or the functions of tumor-infiltrating cells like CD8^+^ T lymphocytes and macrophages.

## Targeting Epigenetics in Melanoma Immunotherapy

Due to the relatively high accessibility and reversibility, epigenetic modification is to some extent easier to be targeted compared to genetic variation, which has brought about some innovations in drug development like the inhibitors of DNMT, HDAC, and EZH2. Based on the mechanistic discoveries of epigenetic regulation of melanoma immunology, multiple clinical trials have been conducted to verify the effect and safety of the combined therapy with both immune checkpoint blockade and epigenetics-targeting drugs, particularly with DNA hypomethylating agents and HDAC inhibition agents that have been documented to play prominent roles in the regulation of antitumor immunity in melanoma.

In terms of DNA hypomethylating agents, the next-generation guadecitabine has gained much attention and has been testified in a clinical trial. Di Giacomo et al. recently reported the progress of the NIBIT-M4 clinical trial, which employs guadecitabine plus ipilimumab to treat patients with unresectable melanoma. Among nineteen patients receiving this combination, the total ir-disease control rate (DCR) and ir-objective response rate (ORR) were 42% and 26%, respectively. The median CpG site methylation of tumor samples at week 4 and week 12 was significantly lower than that of baseline. Notably, the most frequently activated pathways in tissue after the treatment were immune-related ones along with the upregulation of HLA class I on melanoma cells and increased CD8^+^PD-1^+^ T cells and CD20^+^ B cells in posttreatment tumor cores. Overall, this combined therapeutic strategy is safe and tolerable in the treatment of advanced melanoma and has promising immunomodulatory and antitumor activity (132).

Apart from DNA methylation, HDAC is another druggable target to intervene epigenetics and has also been testified in a clinical trial. To be specific, a clinical trial called the PEMDAC study concomitantly employed anti-PD-1 antibody pembrolizumab and HDAC inhibitor entinostat in adult patients with metastatic uveal melanoma (133). Eligible patients had histologically confirmed metastatic uveal melanoma and the primary endpoint was ORR. Patients enrolled in this trial received pembrolizumab 200 mg intravenously every third week in combination with entinostat 5 mg orally once a week. As a result, the ORR of the 29 patients enrolled in this investigation was 14%. The clinical benefit rate at 18 weeks was 28%, with a median progression-free survival of 2.1 months and a median overall survival of 13.4 months. Intriguingly, objective responses and/or prolonged survival were seen in patients with BAP1 wild-type tumors and in one patient with an iris melanoma that exhibited a UV signature. In addition, longer survival was also correlated with lower baseline ctDNA levels or lactate dehydrogenase (LDH). In aggregate, the combination of HDAC inhibition and anti-PD-1 immunotherapy results in durable responses in a subset of patients with metastatic uveal melanoma, with relatively manageable toxicities (134).

Aside from these trials, there are also some other ongoing trials combining epigenetic drugs and immunotherapy in melanoma (**Table 2**). The present applicable DNMT inhibitors also include azacitidine, decitabine, and cedazuridine in addition to the abovementioned guadecitabine. Moreover, three alternative types of HDAC inhibitors, including tinostamustine, domatinostat, and mocetinostat, have also been applied preclinically or clinically aside from the currently available entinostat. For example, in a preclinical tumor model, domatinostat treatment upregulates the expression of antigen-presenting machinery genes and MHC class I and II molecules, along with CTL infiltration in tumors. Domatinostat substantially increases antitumor effects in combination with PD-(L)1 blockade compared to single-agent therapies and displays greater benefit, especially in tumors with preexisting CTLs. Clinically, these findings were confirmed in patients with advanced melanoma treated with domatinostat for 14 days who displayed elevated expression of APM and MHC genes, and the IFN-γ and pembrolizumab response signatures in individual tumor samples. These results highlight the translational potential of the therapeutic approach of combining domatinostat and anti-PD-1 antibody (135). It should be noted that these alternative agents have already been applied to clinical trials in other types of cancer (136). In addition, an EZH2 inhibitor combined with immune checkpoint blockades is also employed in trying to treat urothelial cancer and bladder cancer (136, 137). In the future, more clinical trials are expected to discover additional promising therapeutic combinations based on the regulation of tumor immunology by epigenetics.

**Table 2:** List of clinical trials combining epigenetic drugs and immunotherapy in melanoma.

Clinical trial ID	Recruitment status	Phase	Immunotherapy agent	Epigenetic drug	Cancer type
**DNMTi**					
**NCT02608437**	Completed	I	Ipilimumab	Guadecitabine	Metastatic melanoma
**NCT02816021**	Recruiting	II	Pembrolizumab	Azacitidine	Metastatic melanoma
**NCT05089370**	Not yet recruiting	I/II	Nivolumab	Decitabine/cedazuridine	Mucosal melanoma
**HDACi**					
**NCT02437136**	Unknown	I/II	Pembrolizumab	Entinostat	NSCLC/melanoma, colorectal cancer
**NCT02697630**	Active	II	Pembrolizumab	Entinostat	Uveal melanoma
**NCT03765229**	Recruiting	II	Pembrolizumab	Entinostat	Stage III/IV melanoma
**NCT03903458**	Recruiting	I	Nivolumab	Tinostamustine	Melanoma
**NCT04133948**	Recruiting	I/II	Ipilimumab and nivolumab	Domatinostat	Stage III melanoma
**NCT03565406**	Terminated	I	Ipilimumab and nivolumab	Mocetinostat	Stage III/IV melanoma
**NCT03278665**	Completed	I/II	Pembrolizumab	Domatinostat	Unresectable/metastatic melanoma

NSCLC, non-small cell lung cancer.

## Conclusions

In this review, we highlight the roles of epigenetics in the tumor immunology of melanoma and discuss the innovations of targeting epigenetics combined with immunotherapy from the perspectives of both preclinical mouse models and ongoing human clinical trials. Given that the epigenetic regulations of various paradigms exert a significant regulatory role in not only tumor cell behavior but also the outcome of antitumor immunity, we speculate that targeting epigenetics is a promising therapeutic strategy to achieve the simultaneous effects on both tumor growth and tumor immunology, which warrants more studies in the future.

## Author Contributions

WG and CL designed the review. YC, XY, and NS drafted the manuscript and prepared the figures. WG and CL revised the draft. All authors read and approved the final manuscript.

## Funding

This work has received funding from the National Natural Science Foundation of China (No. 81902791), Support Program of Young Talents in Shaanxi Province (No. 20200303), and Young Eagle Project of Fourth Military Medical University (No. 2019cyjhgwn).

## Conflict of Interest

The authors declare that the research was conducted in the absence of any commercial or financial relationships that could be construed as a potential conflict of interest.

## Publisher’s Note

All claims expressed in this article are solely those of the authors and do not necessarily represent those of their affiliated organizations, or those of the publisher, the editors and the reviewers. Any product that may be evaluated in this article, or claim that may be made by its manufacturer, is not guaranteed or endorsed by the publisher.
